# Photothermal oxidative dehydrogenation of propane to propylene over Cu/BN catalysts

**DOI:** 10.3389/fchem.2024.1439185

**Published:** 2024-07-18

**Authors:** Shaoyuan Sun, Manqi Zhao, Huimin Liu, Dezheng Li, Yiming Lei

**Affiliations:** ^1^ School of Chemical and Environmental Engineering, Liaoning University of Technology, Jinzhou, China; ^2^ Department of Chemistry (Inorganic Chemistry), Faculty of Sciences, Autonomous University of Barcelona (UAB), Barcelona, Spain

**Keywords:** oxidative dehydrogenation of propane, propylene production, photothermal, Cu/BN catalysts, localized surface plasmon resonance

## Abstract

Oxidative dehydrogenation of propane (ODHP) is a reaction with significant practical significance. As for the industrial application of ODHP, it is challenging to achieve high activity and high propylene selectivity simultaneously. In this study, to overcome this obstacle, we designed a series of Cu/BN catalysts with unique morphologies for establishing a photothermal ODHP system with high efficiency and selectivity. Characterization and evaluation results revealed that Cu/BN-NS and Cu/BN-NF with enlarged specific surface areas exhibited higher catalytic activities. The localized surface plasmon resonance (LSPR) effect of Cu nanoparticles further enhanced the photothermal catalytic performances of Cu/BN catalysts under visible light irradiation. To the best of our knowledge, it is the first time to establish a BN-based photothermal ODHP catalytic system. This study is expected to pave pathways to realize high activity and propylene selectivity for the practical application of ODHP.

## 1 Introduction

Oxidative dehydrogenation of propane (ODHP) technology could convert propane in shale gas into propylene (2C_3_H_8_ + O_2_ = 2C_3_H_6_ + 2H_2_O). Propylene is one of the basic raw materials for producing diverse high-value-added chemicals, such as acrylonitrile, propylene oxide, propylene glycol, and epichlorohydrin (Carrero et al., 2014; Chen et al., 2021; Li et al., 2021). Therefore, achieving highly efficient ODHP to obtain propylene has significant practical significance. High temperature is thermodynamically beneficial for ODHP, nevertheless, the desired product propylene from ODHP is prone to be overoxidized at high reaction temperatures, which generally leads to a decrease in its selectivity (Otroshchenko et al., 2021; Fu et al., 2023).

To date, a great number of catalysts have been developed for ODHP, including transition metal oxide catalysts (vanadium-based catalysts, molybdenum-based catalysts, etc.,(Pushkar and Varghese, 2023; Wang et al., 2023), transition metal-based catalysts (such as Pt nanoclusters) (Vajda et al., 2009), rare earth metal oxide catalysts (especially CeO_2_ related catalysts) (Xie et al., 2018) and non-metallic catalysts (B-containing catalysts and C-based catalysts) (Grant et al., 2016; Venegas et al., 2018). Among these catalysts, boron nitride (BN) is a promising candidate for industrial application, since it has high propylene selectivity in ODHP, and exhibits enormous potential for photocatalysis. Therefore, the excellent catalytic performance of BN offers a new path for the selective cleavage of C–H bonds in alkanes (Wu et al., 2008; Love et al., 2019). Unfortunately, the catalytic performance of BN is restricted by insufficient light absorption and rapid electron-hole pairs recombination. What’s more, BN tends to react with the water vapor of the ODHP product under high-temperature conditions, resulting in deactivation (Cao et al., 2023). Considering the aforementioned high-temperature conditions, which not only decrease the selectivity but also limit the carbon neutrality, it is still urgent to further improve the activity of BN in ODHP via optimizing the harsh reaction conditions.

Hermans et al. first applied commercial hexagonal boron nitride (h-BN) and boron nitride nanotubes (BNNTs) as non-metallic catalysts to ODHP reactions, showing excellent catalytic performance, which has attracted extensive attention from researchers, and has achieved fruitful results in research and development in related fields (Grant et al., 2016). Several approaches have been adopted to improve the activity of BN in ODHP, such as increasing the specific surface area of BN, Eswaramoorthy et al. successfully prepared a high specific surface area BN catalyst using boric acid and dicyandiamide reaction, and its specific surface area was increased from 48 m^2^/g to 1,380 m^2^/g compared with commercial BN, significantly increasing the number of active sites (Chaturbedy et al., 2018). Doping BN with foreign elements (Wang G. et al., 2021), Lu’s group prepared a cordierite-supported thin h-BN layer integral catalyst by chemical vapor deposition (CVD) using boric acid and urea as raw materials. The h BN/cordierite catalyst can achieve 16.8% propane conversion and 82.1% propylene selectivity at 535°C and 576,000 mL/(gBN·h) ultra-high air velocity (GHSV) (Wang et al., 2020). Preparing BN with local chemical environment regulated by plasma (Liu Z. et al., 2021). In recent years, photothermal catalytic technology, introducing extra light irradiation into a thermal-driven reaction system, is a prevailing approach to enhance the catalytic performance (Christopher et al., 2011; Linic et al., 2011; Liu et al., 2018). Photothermal catalytic systems have been reported to be capable of boosting the catalytic activity, tuning the selectivity to the target product, and promoting stability, with the assistance of a suitable catalyst (Liu et al., 2015; Song et al., 2019; Liu H. et al., 2021). From this perspective, photothermal catalysis might improve the catalytic activity of BN via accelerating the rate-limiting step and decreasing the activation barrier at mild conditions. To the best of our knowledge, however, there is still no report of establishing a photothermal catalytic ODHP system to promote the performance of BN, because the narrow light response in BN is not enough to induce photothermal effect. Thus, introducing external components is necessary to improve the catalytic properties of BN via photothermal catalysis.

Taking the above-mentioned factors into consideration, in this study, we fabricated a photothermal ODHP catalytic system based on a BN catalyst. In order to introduce a photothermal effect, plasmonic Cu nanoparticles (Cu-NPs) have been loaded onto the BN substrate for utilizing the strong localized surface plasmon resonance (LSPR) effect in Cu-NPs (Kale et al., 2014; Wang et al., 2014; Liu et al., 2024). A series of Cu/BN catalysts with specific structures and plasmonic Cu-NPs have been designed and systematically characterized. The photothermal catalytic ODHP performances of Cu/BN catalysts were evaluated, showing the enhancement impact of unique structures and the photothermal effect in ODHP activity and propylene selectivity. Through investigating a relationship between catalyst properties and catalytic ODHP performances, this work provides guidance for the rational design of economic photothermal catalysts for highly efficient and selective ODHP application.

## 2 Experimental section

### 2.1 Catalyst preparation

BN nanosheet (BN-NS, 99.9%), BN nanofibers (BN-NF, 99.9%), and BN nanoparticles (BN-NP, 99.9%) were purchased and used as supports without further purification or treatment. Cu/BN-NS, Cu/BN-NF, and Cu/BN-NP were prepared by wetness impregnation. In detail, a certain amount of Cu(NO_3_)_2_·3H_2_O (99.0%) was dissolved in deionized water and added into the dispersions containing different BN supports, respectively. After stirring at room temperature at 500 r/min for 10 h, the samples were evaporated and dried at 100°C in air and reduced in a 10% H_2_/N_2_ mixture at 500°C for 3 h (Liu H. et al., 2021). The theoretical loading amounts of Cu on the three Cu/BN catalysts were 1.0 wt%.

### 2.2 Catalyst characterization

The crystalline structures of BN and Cu/BN catalysts were analyzed by X-ray diffraction (XRD) method, on an X-Pert diffractometer equipped with graphite monochromatized Cu-Kα radiation. The specific surface areas were determined with a surface area analyzer (BEL Sorp-II mini, BEL Japan Co., Japan) by the Brunauer-Emmett-Teller (BET) method. The morphology of BN supports and the sizes of Cu particles on Cu/BN catalysts were observed on a transmission electron microscope JEM-2100 (JEOL Ltd., Japan). The diffuse reflection spectra of the catalysts were measured by a UV-visible spectrophotometer (UV-2600, SHIMADZU Co., Japan) from 200 nm to 800 nm. The existences and the valence states of Cu, B, and N over the catalysts were identified by X-ray photoelectron spectroscopy (XPS, PHI Quantera SXM, ULVAC-PHI Inc., Japan).

### 2.3 Catalyst evaluation

ODHP reaction was conducted in a fixed-bed reactor under atmospheric pressure. A 0.10 g portion of the as-prepared catalyst without dilution was packed uniformly in the constant temperature zone of a quartz tube. After pre-reducing the catalysts at 500°C for 2 h (Wang J. et al., 2021), propane and oxygen with a molar ratio of 2/1 were introduced into the reactor at a total flowrate of 20.0 mL min^−1^ (STP), in which the reaction temperature was kept in the range of 400°C–500°C and a 300 W Xe lamp (λ > 420 nm) was employed to provide visible light as an extra light energy source. The effluent gas was analyzed by gas chromatography (GC) to obtain the relative amount of propane, propylene, ethylene, methane, carbon dioxide, and carbon monoxide. The conversion of propane and the selectivity of products were calculated using Eqs 1, 2.

$$C\text{onversion }\left(\%\right)=1‐\frac{C_{\text{propane},\text{out}}}{C_{\text{propane},\text{in}}}\times 100\%$$ (1)

$$\text{Selectivity }\left(\%\right)=\frac{C_{\text{product}}}{\sum C_{\text{products}}}\times 100\%$$ (2)

where C_propane, out_ and C_propane, in_ are the molar flow rates of propane before and after ODHP reaction. C_product_ is the molar flow rate of the products including propylene or other side products after ODHP reaction.

## 3 Results and discussion

### 3.1 Physicochemical properties of BN supports and Cu/BN catalysts

**FIGURE 1:**
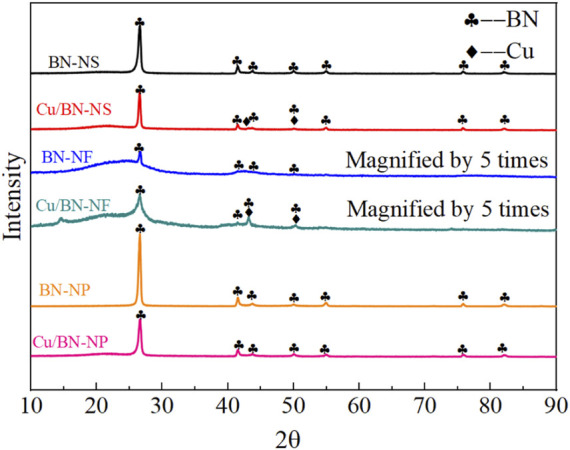
XRD patterns of BN supports and Cu/BN catalysts.

The crystalline structures of different BN supports and the as-prepared Cu/BN catalysts were characterized by XRD as displayed in Figure 1. The characteristic peaks could be seen at 26.8^°^, 41.7^°^, 43.8^°^, 49.9^°^, 54.9^°^, 75.8^°^, and 82.1^°^, which could be assigned to the hexagonal phase of BN (Kostoglou et al., 2015; Liang et al., 2020). Additionally, the diffraction peaks of BN-NP and BN-NS were relatively sharp, indicating that BN-NP and BN-NS were well crystallized. On the contrary, the peak intensity attributed to BN-NF was very low. In order to analyze the crystallinity and phase, the peak intensity was enlarged by 5 times. The magnified peaks of BN-NF were very broad, implying a poor crystallinity of BN-NF. A broad peak appeared in the range of 20°C–30°C, which may be attributed to a small amount of amorphous BN in the BN-NF carrier. An inconspicuous small peak appeared around 15°C, which might be due to the insufficient purity of commercial BN-NF. Over the XRD patterns of Cu/BN catalysts, in addition to the diffraction peaks assigned to the hexagonal phase of BN, another two diffraction peaks at 43.3° and 50.2° appeared over Cu/BN-NS and Cu/BN-NF, which could be attributed to the (111) and (200) facets of metallic Cu, respectively (Figure 1) (Jiang et al., 2015; Gawande et al., 2016) It demonstrated that Cu was successfully loaded onto the supports. Nonetheless, no obvious diffraction peak attributed to Cu was observed over Cu/BN-NP, which might be due to the small sizes of Cu on Cu/BN-NP.

**FIGURE 2:**
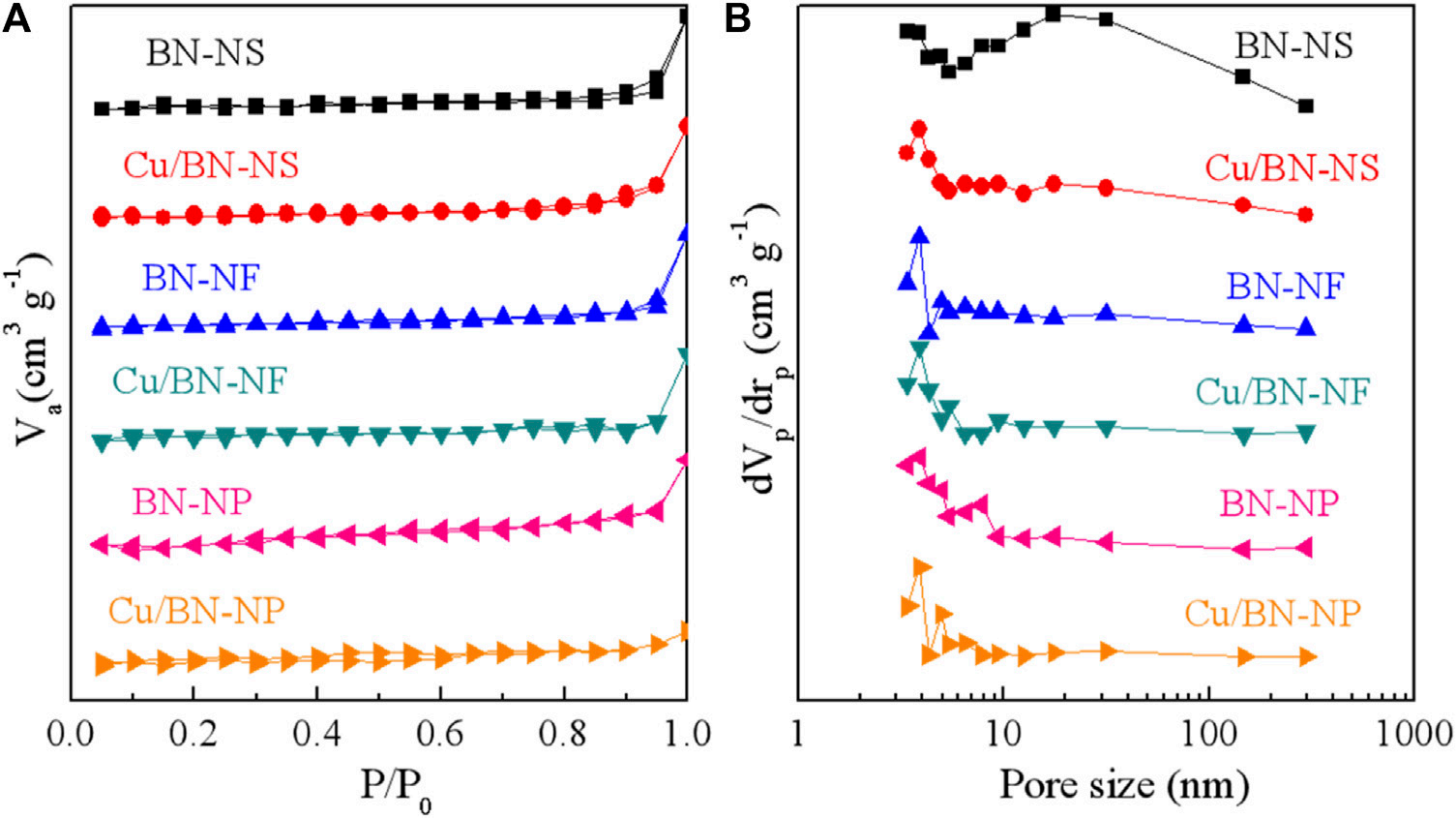
**(A)** Adsorption-desorption isotherms and **(B)** pore size distributions of BN supports and Cu/BN catalysts.

The specific surface areas and pore structures of BN and Cu/BN catalysts were characterized by N_2_ adsorption-desorption characterizations. The adsorption-desorption isotherms and the pore size distributions were shown in Figures 2A, B, respectively. Clearly, all three BN supports adsorption-desorption isotherms exhibited the properties of III-type of adsorption-desorption isotherms, suggesting the possible macropores structures. After loading Cu-NPs, the adsorption-desorption isotherms had almost no changes, indicating that the wetness impregnation process did not changes the pore structure of BN supports (Figure 2A). Indeed, their pore sizes were mainly distributed in the range of 2–10 and 20–100 nm (Figure 2B). The abundant number of pores would be beneficial for the reactant absorption and product desorption during ODHP. The specific surface areas of BN-NS, BN-NF, and BN-NP were 4.6–13.2 m^2^ g^−1^ (Table 1). The low area might be attributed to the accumulation of BN substrates. After loading Cu-NPs, the area of Cu/Bn-NS and Cu/BN-NF had little increase, which could be owing to the exposed Cu-NPs sites. However, the specific area of Cu/BN-NP had a decline. In general, the larger specific surface areas would contribute to the gas-phase reactions as shown in the following section.

**TABLE 1:** Specific surface area (S_BET_), average pore size, and pore volume of BN supports and Cu/BN catalysts extracted from N_2_ adsorption-desorption characterizations, as well as the sizes of Cu on Cu/BN catalysts analyzed from TEM images.

Catalyst	S_BET_ (m^2^·g^−1^)	Average pore size (nm)	Pore volume (cm^3^ g^−1^)	Cu size (nm)
BN-NS	10.3	22.4	1.16 × 10^−1^	—
Cu/BN-NS	16.8	22.4	1.25 × 10^−1^	12.7
BN-NF	13.2	15.0	9.90 × 10^−2^	—
Cu/BN-NF	17.2	13.6	1.17 × 10^−1^	9.7
BN-NP	4.6	5.30	1.20 × 10^−2^	—
Cu/BN-NP	0.06	105.0	2.90 × 10^−3^	7.4

**FIGURE 3:**
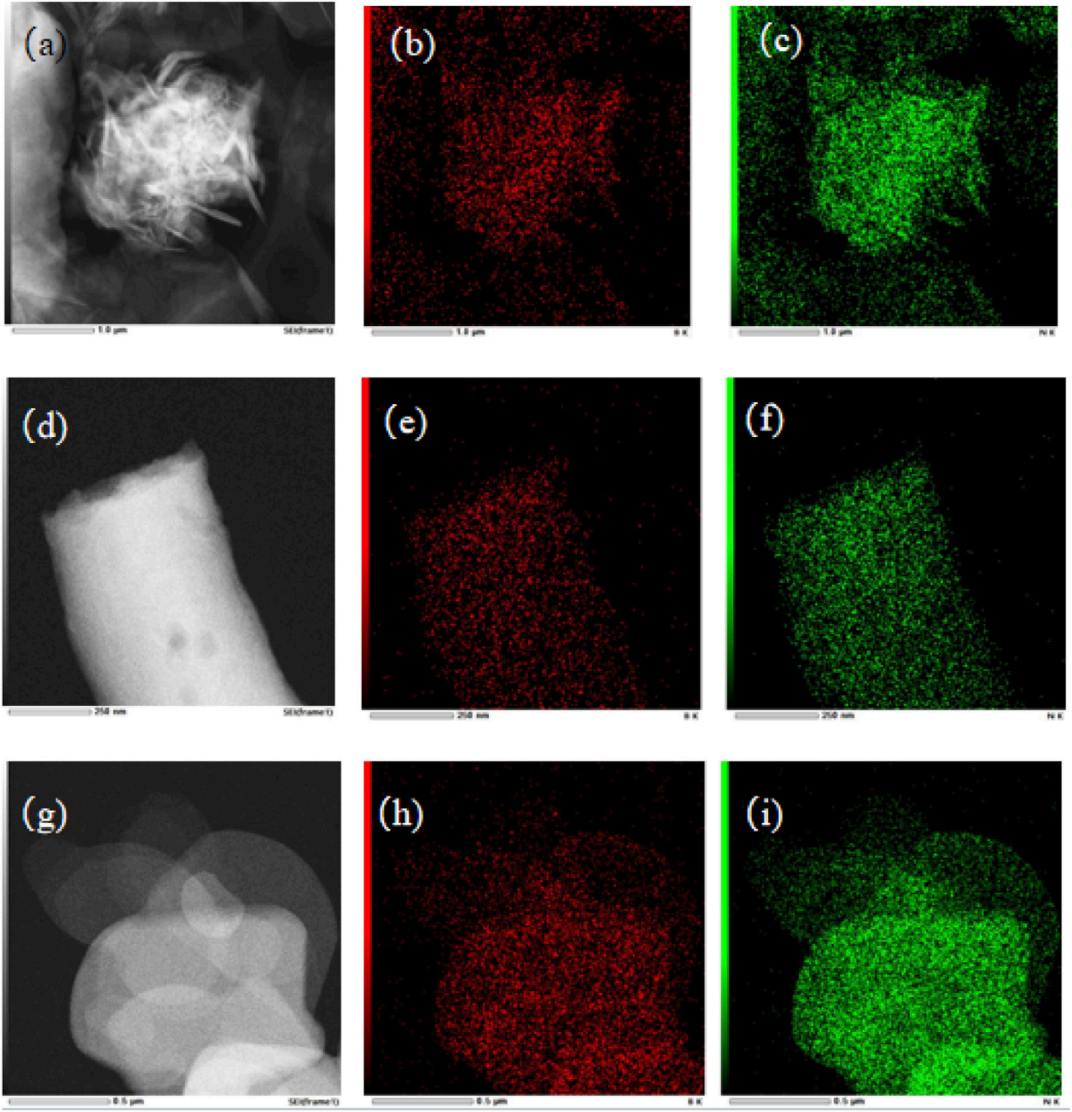
TEM images of **(A)** BN-NS, **(D)** BN-NF and **(G)** BN-NP, and the elemental distributions of B and N over BN-NS: **(B)** and **(C)**; BN-NF: **(E)** and **(F)**; and BN-NP: **(H)** and **(I)**.

The morphologies and elemental distributions of BN-NS, BN-NF, and BN-NP supports were observed via transmission electron microscope (TEM) and energy dispersive spectrometer (EDS) as displayed in Figure 3. Apparently, BN-NS had nanosheet morphologies with sizes varying in the range of 500–1,000 nm (Figure 3A). BN-NF had nanofiber structures with 100–300 nm of diameter (Figure 3D). And BN-NP were of nanoparticle morphologies with an average particle size of around 500 nm (Figure 3G). Over each of the three BN supports, the elemental distributions of B and N indicated the existence of B and N elements in BN-NS, BN-NF, and BN-NP supports (Figures 3B, C, E, F, H, I) (Lin and Connell, 2012; Zhou et al., 2018)

**FIGURE 4:**
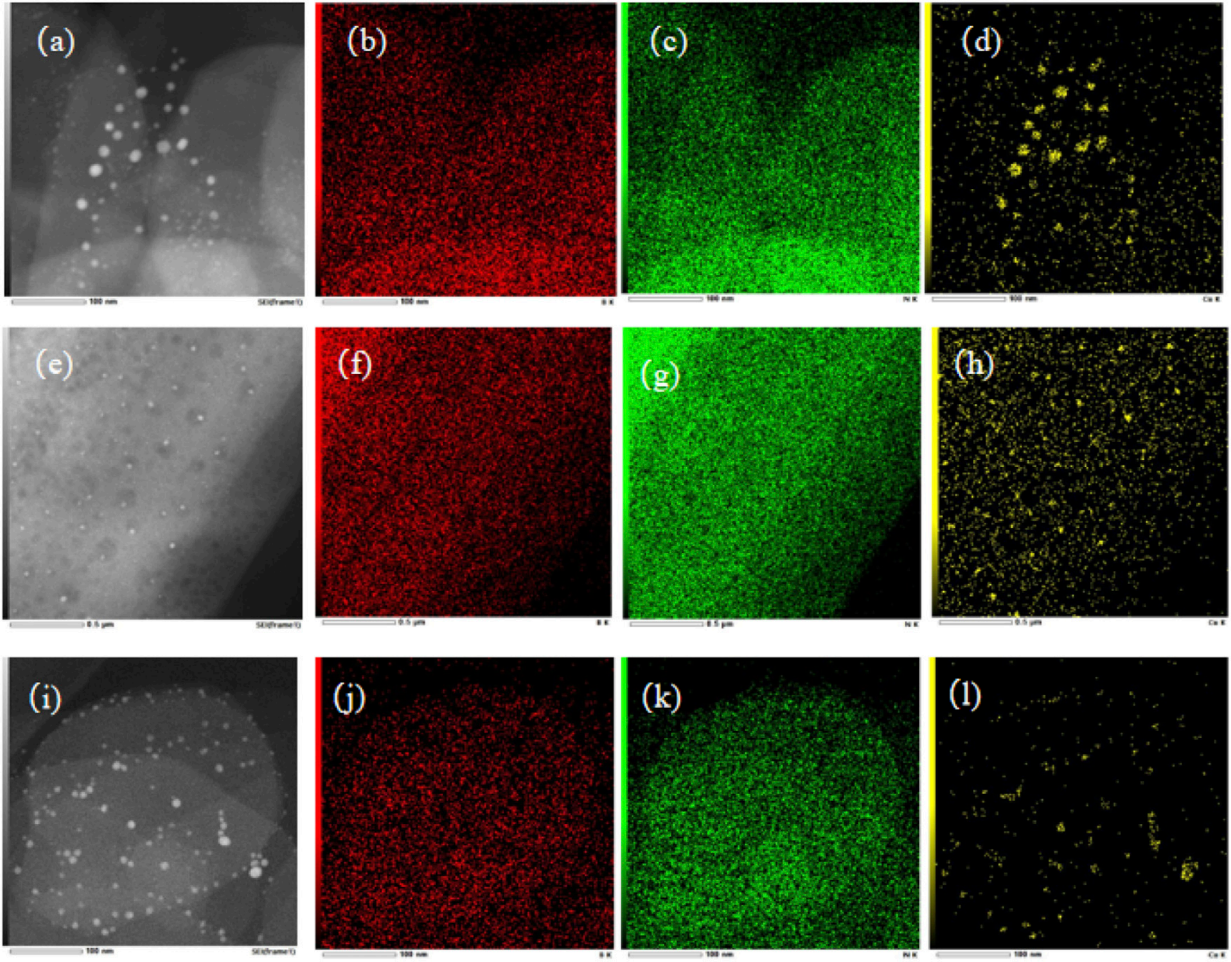
TEM images of **(A)** Cu/BN-NS, **(E)** Cu/BN-NF, and **(I)** Cu/BN-NP, and the elemental distributions of B, N and Cu over Cu/BN-NS: **(B)**, **(C)**, and **(D)**; Cu/BN-NF: **(F)**, **(G)** and **(H)**; and Cu/BN-NP **(J)**, **(K)**, and **(L)**.

The morphologies of Cu/BN catalysts were further characterized via TEM, with their images and elemental distributions of Cu, B, and N shown in Figure 4. Obviously, the morphologies of BN on Cu/BN catalysts remained the same as their corresponding support counterparts (Figures 4A, E, I), indicating the structure of BN was not destroyed during the catalyst preparation procedure. In addition, small dark spots were observed over the TEM images of the three Cu/BN catalysts. Elemental mapping suggested that the dark spots were Cu-NPs, which confirmed the successful loading of Cu onto BN supports (Figures 4D, H, L). The average sizes of Cu nanoparticles on the three catalysts were analyzed, and they were 12.7, 9.7, and 7.4 nm on Cu/BN-NS, Cu/BN-NF, and Cu/BN-NP, respectively (Table 1). Moreover, over each of the three Cu/BN catalysts, the co-exhibition of B and N elements in Cu/BN catalysts could also be detected similar to their corresponding pristine BN supports (Figures 4B, C, F, G, J, K).

**FIGURE 5:**
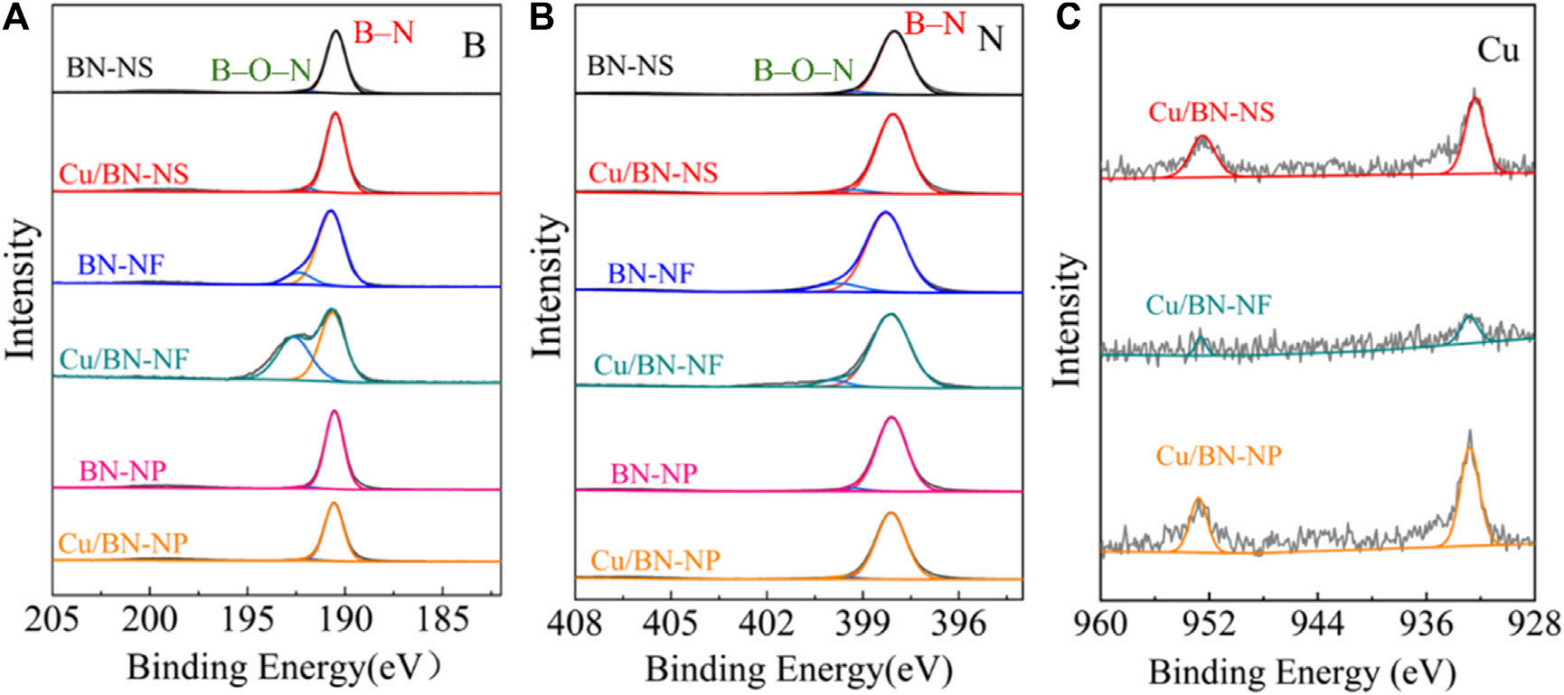
XPS spectra of **(B) (A)**, N: **(B)**, and Cu: **(C)** over BN supports and Cu/BN catalysts.

The elemental valence states of B and N were characterized via XPS. All three BN supports showed the standard deconvolution peaks of BN. The binding energies of B were located at 190.2 eV and 192.6 eV (Figure 5A), which should be attributed to the B–N and B–O–N chemical bonds, respectively. The binding energies of N were centered at 398.1 eV and 400.2 eV (Figure 5B), similarly, which could be assigned to B–N and B–O–N binding, respectively (Wu et al., 2016; Jiang et al., 2018; Liang et al., 2020). The presence of B–O–N characteristic peaks was the results of inevitable surface oxidation, which always occurred in BN materials. From Figure 5, the binding energies of B and N over Cu/BN-NS and Cu/BN-NP catalysts were the same as their corresponding support counterparts, indicating the valence states of B and N were not changed after the loading of Cu (Figures 5A, B). On the contrary, over the Cu/BN-NF catalyst, compared to the support BN-NF, the intensity of the peak attributed to B at 190.2 eV was shortened and the peak at 192.6 eV was sharpened (Figure 5A); whereas the peak of N centered at 398.1 eV was bigger and the peak at 400.2 eV was smaller over Cu/BN-NF catalyst (Figure 5B). It indicated the electron coupling between Cu-NPs and BN-NF. In addition, the peaks at 932.8 eV and 952.5 eV could be observed over the Cu 2p spectra of the Cu/BN catalysts (Figure 5C), which belong to Cu 2p_3/2_ and Cu 2p_1/2_ (Liang et al., 2020; Liang et al., 2022). It further confirmed the successful loading of Cu onto BN supports.

### 3.2 Optical properties of BN supports and Cu/BN catalysts

**FIGURE 6:**
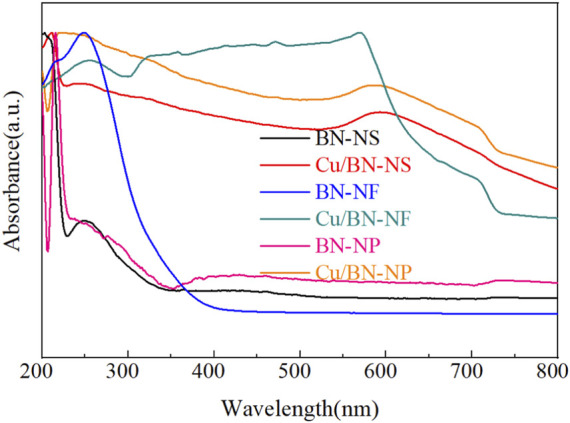
UV-vis spectra of BN supports and Cu/BN catalysts.

The light-harvesting capacities of BN supports and Cu/BN catalysts were characterized by UV-vis spectroscopy as shown in Figure 6. The light absorption edge of three pristine BN supports was mainly located at UV light range with a wavelength shorter than 400 nm (Figure 6). After the loading of Cu, the light absorption capacities of Cu/BN catalysts were extended to visible light and even near-infrared light regions (Figure 6). Obvious absorption peaks of Cu/BN-NS, Cu/BN-NF, and Cu/BN-NP in the range of 550–600 nm could be observed, which could be attributed to the localized surface plasmon resonance (LSPR) effect of Cu-NPs (Wang et al., 2005; Yao et al., 2020). It demonstrated that the plasmonic Cu-NPs could enhance the light-harvesting capacities, especially in the visible light region, which was a requirement for inducing photothermal effect.

### 3.3 (Photo)thermal catalytic ODHP performances of Cu/BN catalysts

**FIGURE 7:**
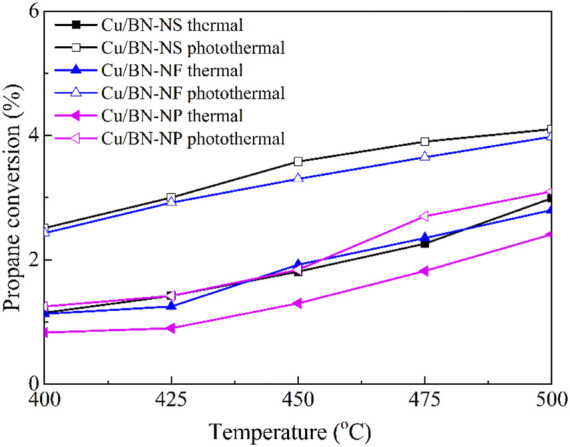
Catalytic activities of Cu/BN catalysts in traditional thermal-driven catalytic system and photothermal catalytic ODHP system. Reaction conditions: 0.10 g catalyst, pre-reduced at 500°C for 2 h, propane: oxygen = 2: 1, total flowrate 20.0 mL min^−1^, reaction temperature 400–500°C, with and without a 300 W Xe lamp irradiation (λ > 420 nm).

Once confirming the successful synthesis of Cu/BN catalysts and the fundamental properties involving mesoporous structures, electron coupling, and enhanced light absorption abilities for improving catalytic activity, the catalytic performances of Cu/BN catalysts in ODHP were evaluated in a fixed-bed reactor under the traditional thermal-driven reaction and photothermal catalytic systems. The activities of the three Cu/BN catalysts were displayed in Figure 7. Clearly, under the traditional thermal-driven reaction system, the catalytic activities of the Cu/BN catalysts followed the trend of Cu/BN-NS ≈ Cu/BN-NF > Cu/BN-NP. At 400°C temperature, Cu/BN-NS and Cu/BN-NF recorded a propane conversion of ∼1.1%, while Cu/BN-NP gave a propane conversion of only ∼0.8%. The higher activities of Cu/BN-NS and Cu/BN-NF might be due to their relatively larger specific surface area, which exposed more active sites for reactant adsorption and subsequent reaction procedure (Chaturbedy et al., 2018). With the increase in reaction temperature, the catalytic activities of all three catalysts were improved Cu/BN-NS and Cu/BN-NF recorded a propane conversion of ∼3% and ∼2.8% respectively, while Cu/BN-NP gave a propane conversion of ∼2.4%, when the temperature reaches 500°C. Which since the high temperatures often favored propane conversion as mentioned above (Figure 7). Under an as-designed photothermal catalytic system, the activities of all three catalysts were further enhanced owing to the photothermal mechanism in the LSPR effect of Cu-NPs under visible light irradiation (Figure 7). (Wang et al., 2005; Yao et al., 2020) Notably, Cu/BN-NS afforded the highest photothermal catalytic activity in ODHP, reaching 4.1% at 500°C. while Cu/BN-NF and Cu/BN-NP recorded a propane conversion of ∼3.9% and ∼3.1% respectively.

**FIGURE 8:**
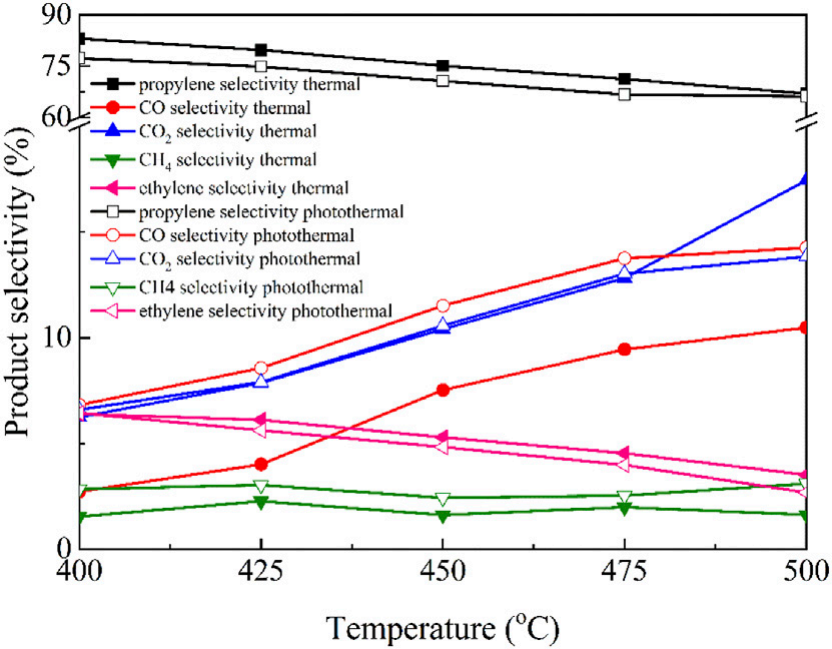
Catalytic selectivities of Cu/BN-NS in traditional thermal-driven catalytic system and photothermal catalytic ODHP system. Reaction conditions: 0.10 g catalyst, pre-reduced at 500°C for 2 h, propane: oxygen = 2: 1, total flowrate 20.0 mL min^−1^, reaction temperature 400–500°C, with and without a 300 W Xe lamp irradiation (λ > 420 nm).

The product selectivity of Cu/BN-NS in thermal and photothermal ODHP processes was evaluated as shown in Figure 8. Obviously, in addition to the desired product propylene, a series of by-products including CO, CO_2_, CH_4,_ and ethylene were also produced because of the competitive or overoxidation reactions. With the increase of reaction temperatures from 400°C to 500°C, under both thermal-driven or photothermal catalytic systems, the selectivity towards propylene and ethylene was decreased. On the contrary, the selectivity towards CO and CO_2_ was increased under the same conditions. However, there were only slight changes in the selectivity towards CH_4_. Moreover, compared with the traditional thermal-driven reaction conditions, the selectivity towards propylene and ethylene was slightly lower under the photothermal catalytic system, on the contrary, the selectivity towards CO, CO_2,_ and CH_4_ was higher under the photothermal catalytic system.

**FIGURE 9:**
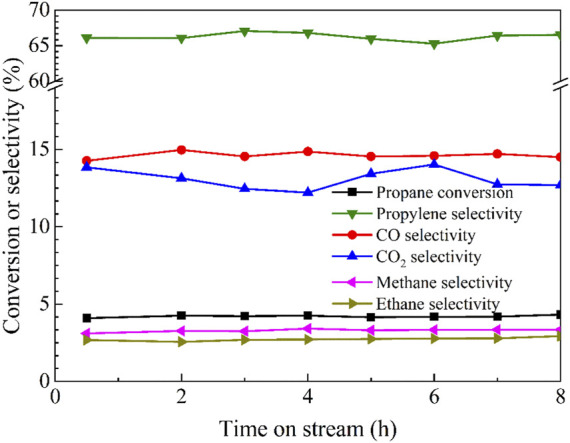
Stability test of Cu/BN-NS in photothermal catalytic ODHP system. Reaction conditions: 0.10 g catalyst, pre-reduced at 500°C for 2 h, propane: oxygen = 2: 1, total flowrate 20.0 mL min^−1^, reaction temperature 500°C with a 300 W Xe lamp irradiation (λ > 420 nm).

Regarding the best catalytic performance of Cu/BN-NS in photothermal ODHP at 500°C, its stability test was conducted under the same conditions. The results in Figure 9 revealed that propane conversion and product selectivity were maintained for 8 h. It suggested that Cu/BN-NS had moderate stability in photothermal ODHP. A comparison table has been summarized to display the advantages of the as-designed Cu/BN-NS catalyst in conversion efficiency and selectivity (Table 2).

**TABLE 2:** Comparison table of thermal and photothermal catalysts for ODHP reactions.

Catalysts	T (°C)	C_3_H_8_ conv. (%)	C_3_H_6_ selec. (%)	Ref.
Cu/BN-NS	500	4.1	68.5	This work
RGO-2%ND	400	2.5	70.0	Roldán et al. (2015)
Cr_3.4_/Al_2_O_3_	550	3.3	92.9	Takahara et al. (1998)
Bulk hBN	500	3	87.0	Grant et al. (2016)
V_2_O_5_/graphene	475	4.7	92.4	Fattahi et al. (2014)
C_r2.0_AlBeta	550	4.5	45.1	Michorczyk et al. (2020)
V-ZrO_2_	400	4	64.6	Ternero-Hidalgo et al. (2019)
0.98V/SiO_2_	525	3.8	68	Barman et al. (2016)
MIL-100(Fe)	120	1.7	46.7	Simons et al. (2019)

## 4 Conclusion

In this study, a photothermal catalytic ODHP system based on a series of Cu/BN catalysts was designed with the aim to achieve enhanced activity and propylene selectivity simultaneously. Under the traditional thermal-driven reaction system (500°C), Cu/BN-NS, Cu/BN-NF, and Cu/BN-NP showed a propane conversion of ∼3% ∼2.8%, and ∼2.4% respectively. The catalytic activities of the Cu/BN catalysts followed the trend of Cu/BN-NS > Cu/BN-NF > Cu/BN-NP, which is in accordance with the specific surface areas of these catalysts. With the illumination of visible light, the catalytic performances of all three Cu/BN catalysts were promoted. Cu/BN-NS、Cu/BN-NF and Cu/BN-NP recorded a propane conversion of ∼4.1%, ∼3.9% and ∼3.1% respectively. The results confirmed the enhancement role of the LSPR effect of Cu-NPs in propane conversion efficiency via inducing a photothermal effect. Considering the fact that this is the first report about a BN-based photothermal ODHP system with a LSPR effect, our study is expected to provide guidance for the rational design of efficient plasmonic-assisted BN-based catalysts used for photothermal ODHP implementation.

## Data Availability

The original contributions presented in the study are included in the article/Supplementary Material, further inquiries can be directed to the corresponding authors.
